# Influence of body mass index and waist–hip ratio on male semen parameters in infertile men in the real world: a retrospective study

**DOI:** 10.3389/fendo.2023.1148715

**Published:** 2023-06-26

**Authors:** Shuxian Wang, Baorui Wu, Changming Wang, Zongpan Ke, Ping Xiang, Xuechun Hu, Jun Xiao

**Affiliations:** ^1^ Changzhou Maternal and Child Health Care Hospital, Changzhou Medical Center, Nanjing Medical University, Nanjing, Jiangsu, China; ^2^ Department of Urology, Division of Life Sciences and Medicine, The First Affiliated Hospital of USTC, University of Science and Technology of China, Hefei, Anhui, China

**Keywords:** body mass index, waist-hip ratio, semen parameters, testosterone, follicular stimulating hormone

## Abstract

**Background:**

It is suggested that body mass index (BMI) can affect male semen quality; however, the results remain controversial. In addition, most studies have focused on the effect of obesity on semen quality. Evidence on the relationship of underweight or waist-hip ratio (WHR) with semen quality is rare. This study aimed to assess the association of BMI and WHR with semen quality.

**Methods:**

Data, including BMI and WHR, was collected from 715.00 men who underwent a fertility evaluation. BMI (kg/m^2^) was categorized as <18.50 (underweight), 18.50–24.90 (normal), 25.00–27.90 (overweight), and ≥28.00 (obese) kg/m^2^ for analysis. WHR was categorized as <0.81 (normal) and ≥0.81 (high). Semen volume, sperm concentration, progressive motility, and total motile sperm count were detected by experienced clinical technicians.

**Results:**

Spearman’s correlation showed that BMI was weakly associated with sperm progressive motility (r = 0.076, *P* < 0.05), while WHR showed no relationship with semen parameters. The azoospermia rate was significantly higher (33.33% vs. 2.10%, *P* < 0.001) and the sperm concentration was lower (*P* < 0.05) in the underweight group. The nonlinear correlation analysis showed that BMI was negatively associated with sperm concentration while BMI was more than 22.40 kg/m^2^ (*P* < 0.05), while WHR was negatively related to sperm progressive motility within 0.82 to 0.89 (*P* < 0.05). Furthermore, the multivariate logistic analysis showed that follicular stimulating hormone (FSH) was an independent risk factor for normal sperm concentration (odds ratio [OR]: 0.791, *P* = 0.001) and morphology (OR: 0.821, *P* = 0.002), BMI was an independent risk factor for normal sperm progressive motility, and testosterone was an independent risk factor for sperm morphology (OR: 0.908, *P* = 0.023).

**Conclusion:**

BMI and WHR were significantly associated with semen parameters, while BMI was an independent risk factor for normal sperm progressive motility. Reproductive hormones, including FSH and testosterone, had a significant influence on sperm concentration and sperm morphology.

## 1 Introduction

Infertility is a global clinical concern, affecting 10.00–15.00% of reproductive-age couples. It is believed that 40% of cases are due to male factors (1–3). Poor semen quality is the major condition leading to male infertility. Although studies have reported several risk factors—including environmental pollutants, Mumps virus infection, and alcohol intake—that may be related to decreased semen quality, the underlying causes are still uncertain (4–6).

Recently, an increasing number of studies have explored the relationship between abnormal body mass index (BMI) and semen quality; however, the results have remained controversial. For example, Michael et al. (7) suggested that an increased BMI and waist circumference (WC) were associated with a reduction in ejaculate volume and the total sperm count, but no relationship was found between BMI and sperm concentration, sperm motility, sperm vitality, sperm morphology, or the DNA fragmentation index in the United States. Wang et al. (8) reported that an increased BMI was linked with a lower total sperm number and sperm concentration in northern China. Meanwhile, another large single-center clinical study by Ma et al. found that being underweight or overweight were both factors associated with a decreased total motile sperm count (9). On the right hand, the researchers found that being overweight was related with a reduction in semen volume and total sperm number, while no correlation with sperm concentration was observed, which was consistent with the study by Michael et al. (7, 9). In addition, Lu et al. (10) found that BMI, the waist–hip ratio (WHR), and WC cannot predict male semen quality; however, semen quality was significantly related to levels of follicular stimulating hormone (FSH) and luteinizing hormone (LH).

Taken together, the evidence on the relationship between body size (BMI or WHR) and semen quality is limited and inconclusive. In the present study, we performed a retrospective study of 715 healthy sperm donors to assess the association of BMI and WHR with semen quality.

## 2 Methods

### 2.1 Study design

A total of 715, male partners of infertile couples who could not achieve pregnancy after 12, consecutive months without contraception at the Reproductive and Genetic Hospital, Division of Life Sciences and Medicine, The First Affiliated Hospital of USTC, University of Science and Technology of China, were recruited for our study. The experimental study protocol was administrated by the Research Ethics Committee of The First Affiliated Hospital of USTC, University of Science and Technology of China, and informed consent was obtained from all participants. Semen samples from the patients with azoospermia were analyzed at least twice at an interval of 3 weeks according to the World Health Organization (WHO) Laboratory Manual for the Examination and Processing of Human Semen (5th edition). Participants were divided into four groups by different BMI values: <18.50 (underweight), 18.50–24.90 (normal), 25.00–27.90 (overweight), and ≥28.00 (obese) kg/m2 (11).WHR was categorized into two groups: <0.81 (normal) and ≥0.81 (high) (12). The patients with a history of cryptorchidism, varicocoele or testicular trauma, administration of hormones, genital infections, or other diseases during the previous 3.00 months were excluded.

### 2.2 Semen and hormone analysis

The semen volume was detected by the weighing method according to the WHO guidelines (2010). Computer-assisted sperm analysis was used to measure sperm concentration, progressive motility, and total motility (SAS, Beijing, China). Sperm morphology was determined through Diff-Quick staining (Anke Biotechnology, Hefei, China). The result of semen analysis including oligospermia, asthenospermia and teratospermia were undertaken according to the WHO Semen Manual, 5th edition. In detail, oligospermia was defined as the sperm concentration <15.00 * 10^6/ml, asthenospermia as P R< 32.00%, teratospermia as normal morphology of spermatozoa < 4.00% while azoospermia was defined as the absence of spermatozoa in the semen. Blood samples were obtained at 8–11 a.m. and were centrifuged for 10 min at 1800 g. The levels of testosterone, LH, and FSH were determined by radioimmunoassay (Beckman Coulter, Brea, USA).

### 2.3 Statistical analysis

All data were evaluated for the normal distribution by the Kolmogorov–Smirnov test. The variables departing from the normal distribution were summarized as medians and interquartile intervals. Correlations between BMI, WHR, and semen parameters were analyzed by Spearman’s correlation coefficient, as appropriate. A one-way ANOVA or Kolmogorov–Smirnov test was used to evaluate the differences among the groups. Univariate and multivariate logistic analysis was performed to seek the independent risk factors for semen parameters. Statistical analyses were performed using IBM SPSS 26 for windows and *P* value < 0.05 was considered statistically significant. The non-linear relationship between BMI, WHR, and semen quality were analyzed by the restricted cubic spline.

## 3 Results

### 3.1 Characteristics of studied men and correlation analysis

The basic data of the participants are shown in **Table 1**. BMI, WHR, and semen parameters were non-normally distributed, which were presented as medians and interquartile intervals. The correlations of BMI and WHR with semen parameters are shown in **Table 2**; a weak positive correlation was found between BMI and sperm progressive motility while both BMI and WHR were negatively correlated with serum testosterone. Other semen parameters, such as total motile sperm count (TMSC) and acrosome integrity, were not related to BMI or WHR.

**Table 1:** Characteristics of men in the study population.

Variables	Mean ± SD or Median (25%-75% quartiles)	Total N
Age (years)	29 (26-31)	715
BMI (kg/m^2)	23.63 (21.88-25.61)	715
WHR	0.85 (0.81-0.89)	715
Time of abstinence (days)	4 (3-6)	715
Semen volume (ml)	3.20 (2.50-4.00)	715
Sperm concentration (×10^6/ml)	54.08 (29.47-93.68)	715
Total sperm count (×10^6/ml)	176.51 (89.50-296.67)	715
Progressive motility (a+b, %)	31.33 (21.70-39.35)	715
Normal morphology rate (%)	5.33 (4.07-6.33)	715
TMSC (×10^6)	2.56 (0.99-5.32)	715
Acrosome integrity (%)	56.62 (50.46-60.62)	715
Anti-sperm antibody	92 (12.87%)	715
FSH (IU/l)	4.82 (3.66-5.94)	170
T (nmol/l)	14.12 (11.61-17.83)	151
LH (IU/l)	4.64 (3.28-6.36)	170
Alcohol	444 (62.10%)	715
Smoking	316 (44.20%)	715

BMI, Body Mass Index; WHR, Waist-Hip Ratio; TMSC, Total Motile Sperm Count; FSH: Follicular Stimulating Hormone; LH, Luteinizing Hormone; T, Testosterone.

**Table 2:** Correlations of BMI and WHR with semen parameters, serum hormones.

Variables	BMI (kg/m2)	*P*	WHR	*P*
Sperm concentration (×106/ml)	-0.035	0.347	-0.021	0.576
Total sperm count (×106/ml)	-0.025	0.497	-0.028	0.449
Semen volume (ml)	0.019	0.605	-0.025	0.511
Progressive motility (a+b, %)	0.076	**0.042**	0.036	0.340
Normal forms (%)	0.044	0.241	0.030	0.428
TMSC (*10^6)	0.016	0.670	0.010	0.794
Acrosome integrity (%)	0.042	0.257	0.059	0.116
Anti-sperm antibody	-0.062	0.095	-0.027	0.477
FSH (IU/l)	-0.073	0.346	-0.149	0.052
T (nmol/l)	-0.469	**<0.001**	-0.484	**<0.001**
LH (IU/l)	-0.046	0.555	-0.170	0.027

TMSC, Total Motile Sperm Count; FSH, Follicular Stimulating Hormone; LH, Luteinizing Hormone; T, Testosterone. Statistically significant data were represented in bold.

### 3.2 Comparison of semen parameters in different groups divided by BMI and WHR

**Figure 1:**
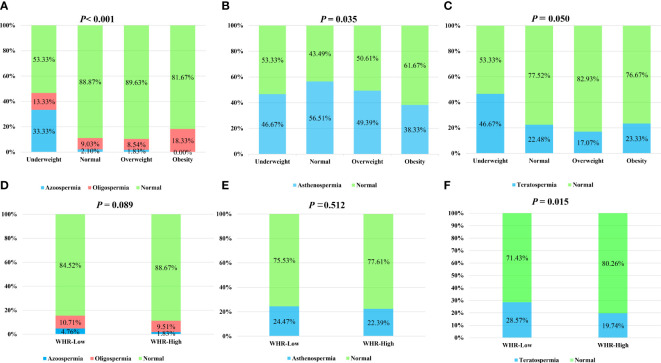
Chi-square test to analyze the influence of body mass index (BMI) and waist–hip ratio (WHR) on the sperm concentration, sperm progressive motility, and sperm morphology. BMI was categorized as: underweight (<18.0 kg/m^2^), normal weight (18.0–24.9kg/m^2^), overweight (25–27.9 kg/m^2^), and obese (≥28 kg/m^2^). WHR was categorized into two groups: <0.81 (normal) and ≥0.81 (high). **(A)** The rate of azoospermia, oligospermia, and normal sperm concentration in the different BMI groups. **(B)** The rate of asthenospermia and normal sperm progressive motility in the different BMI groups. **(C)** The rate of teratospermia and normal sperm morphology in the different BMI groups. **(D)** The rate of azoospermia, oligospermia, and normal sperm concentration in the different WHR groups. **(E)** The rate of asthenospermia and normal sperm progressive motility in the different WHR groups. **(F)** The rate of teratospermia and normal sperm morphology in the different WHR groups.

**Figure 2:**
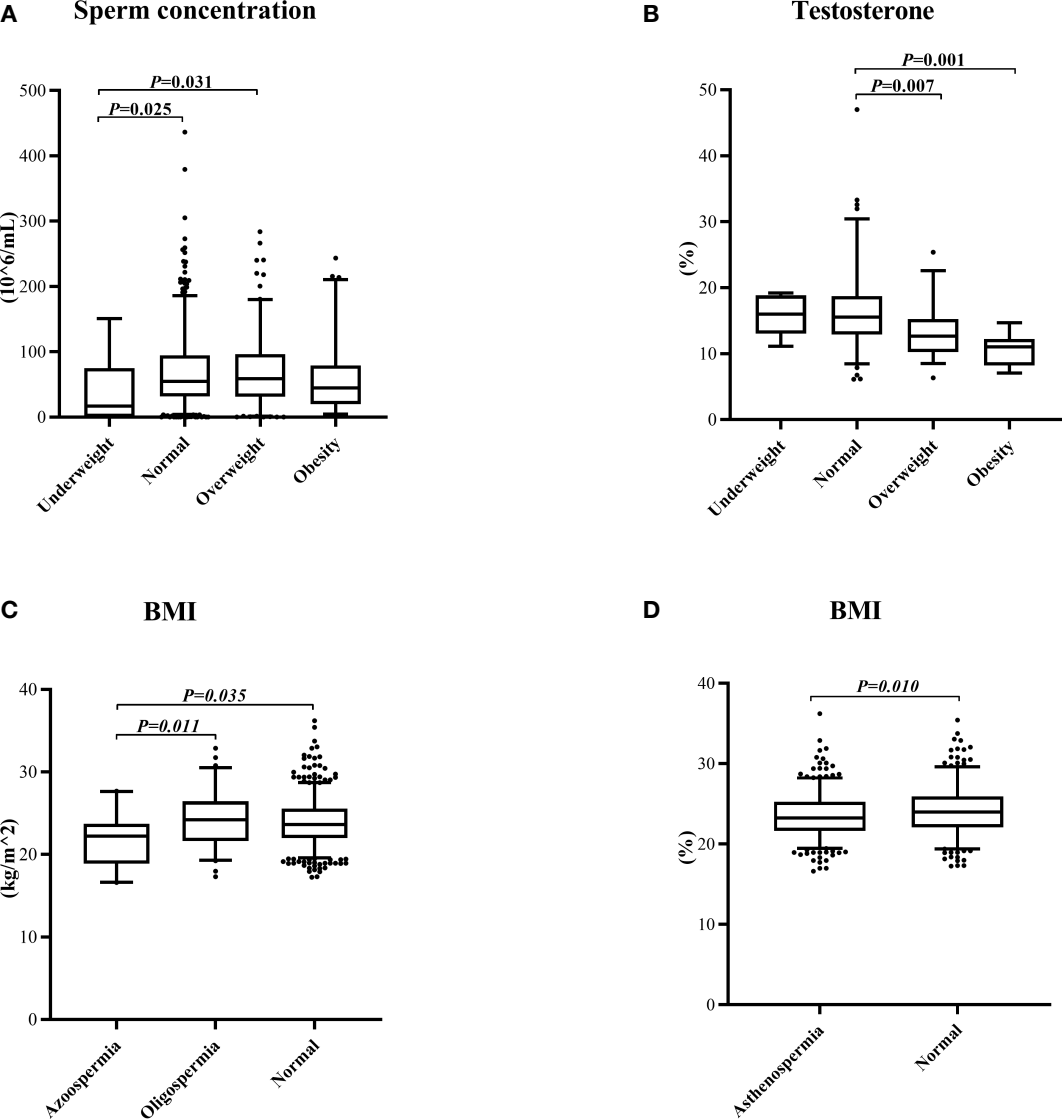
Kolmogorov–Smirnov test to evaluate the differences among the groups. **(A)** The sperm concentration in the different body mass index (BMI) groups. **(B)** The testosterone concentration in the different BMI groups. **(C)** The BMI in the different sperm concentration groups. **(D)** The BMI in different sperm progressive motility groups.

Firstly, the prevalence of oligospermia, asthenospermia, and teratospermia in each group was compared by the chi-square test. There was a reduction of the normal sperm concentration rate in the underweight group compared with other groups (**Figure 1A**). Interestingly, the prevalence of asthenospermia was higher in the normal BMI group and a higher rate of teratospermia was found in the normal WHR group (**Figures 1B, F**). However, no difference was found in the rate of teratospermia in different BMI groups while both the rate of oligospermia and asthenospermia showed no statistically significant differences in the different WHR groups (**Figures 1C–E**). Furthermore, the Kolmogorov–Smirnov test was used to evaluate the differences among each group. The sperm concentration in the underweight group was significantly lower than the normal BMI group and overweight group. No difference was found in any other group (**Figure 2A**). Furthermore, the level of serum testosterone did not show a consistent change; serum testosterone in the overweight group and obese group were significantly higher than in the normal BMI group (**Figure 2B**). In addition, we found that the BMI level in the azoospermia group was lower compared with the oligospermia group and normal sperm concentration group (**Figure 2C**). The BMI was lower in the asthenospermia group than the normal sperm motility group (**Figure 2D**).

### 3.3 Restricted cubic spline analysis of the nonlinear correlation of BMI and WHR with sperm concentration, sperm morphology, and sperm progressive motility

**Figure 3:**
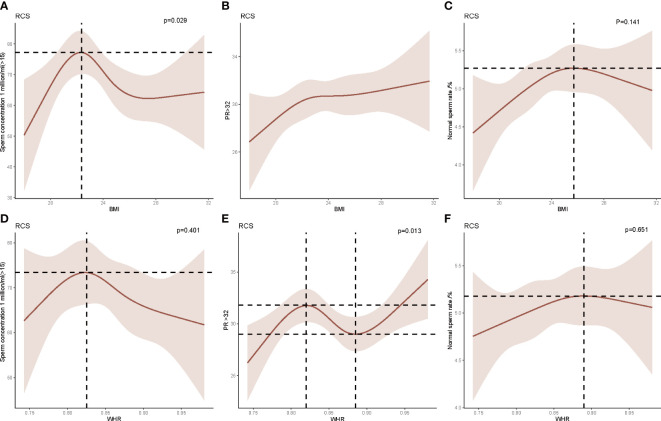
Restricted cubic spline (RCS) analysis of the relationship between body mass index (BMI), waist–hip ratio (WHR), and sperm concentration, sperm progressive motility, and sperm morphology. **(A)** The relationship between BMI and sperm concentration. **(B)** The relationship between BMI and sperm progressive motility. **(C)** The relationship between BMI and sperm morphology. **(D)** The relationship between WHR and sperm concentration. **(E)** The relationship between WHR and sperm progressive motility. **(F)** The relationship between WHR and sperm morphology.

The nonlinear correlation between BMI and semen quality parameters was expressed in **Figures 3A–C**, while **Figures 3D–F** shows the nonlinear correlation between WHR and semen quality. The result indicated that when the BMI was lower than the median BMI in normal weight subjects (22.40 kg/m^2^), the sperm concentration appeared to increase monotonically with increasing BMI. The sperm concentration was inversely associated with increasing BMI While BMI was more than 22.4 kg/m^2^. No significant relationships were observed between BMI and sperm progressive motility or sperm morphology. Interestingly, the sperm progressive motility was positively associated with the WHR when <0.82 or >0.89 and negatively associated with the WHR within 0.82 to 0.89. No relationship was observed between the WHR and sperm concentration or sperm morphology.

### 3.4 Multivariate logistic analysis of the independent risk factors for sperm concentration, sperm progressive motility, and sperm morphology

The odds ratios (ORs), 95% confidence interval (CIs), and *P* values of the sperm concentration, sperm morphology, and sperm progressive motility are summarized in **Tables 3**–**5**. The results of the ordinal multivariate logistic analysis revealed that age (OR: 1.184, 95% CI: 1.015–1.381, *P* = 0.031) and FSH (OR: 0.791, 95% CI: 0.692–0.904, *P* =0.001) were independent risk factors of male sperm concentration; while BMI, WHR, serum testosterone, LH, alcohol and smoke intake were not. Interestingly, referring to sperm motility, the results of the multivariate logistic analysis showed that BMI (OR: 1.072, 95% CI: 1.019–1.128, *P* = 0.007) and abstinence time (OR: 0.913, 95% CI: 0.853–0.978, *P* = 0.009) were the only independent risk factors among the clinical parameters counted. Finally, the multivariate logistic analysis suggested that FSH (OR: 0.821, 95% CI: 0.727–0.927, *P* = 0.002) and testosterone (OR: 0.908, 95% CI: 0.835–0.987, *P* = 0.023) were the independent risk factors for sperm morphology, while BMI or WHR were not found to be associated with it.

**Table 3:** Univariate and multivariate ordinal logistic analysis for screening the independent factors of sperm concentration.

Variables	OR	Univariate analysis	*P*	B	OR	Multivariate analysis	*P*
		95%CI				95%CI	
Age (years)	1.051	0.674-1.105	**0.05**	0.169	1.18	1.015-1.381	**0.031**
BMI (kg/m^2^)	1.008	0.934-1.087	0.84				
WHR	1.489	0.028-80.238	0.84				
Time of	1.040	0.941-1.149	0.44				
Anti-sperm	1.242	1.234-9.718	**0.01**	1.095	2.98	0.337-	0.325
FSH (IU/l)	0.757	0.668-0.858	**<0.0**	–	0.79	0.692-0.904	**0.001**
T (nmol/l)	0.953	0.8841-1.028	0.21				
LH(IU/l)	0.800	0.712-0.899	**<0.0**	–	0.88	0.783-1.001	0.053
Alcohol	1.150	0.846-1.564	0.37				
Smoke	0.707	0.452-1.105	0.12				

BMI, Body Mass Index; WHR, Waist-Hip Ratio; FSH, Follicular Stimulating Hormone; LH, Luteinizing Hormone; T, Testosterone. Statistically significant data were represented in bold.

**Table 4:** Univariate and multivariate logistic analysis for screening the independent Influencing factors of sperm progressive motility.

Variables	OR	Univariate analysis	*P*	B	OR	Multivariate analysis	*P*
		95%CI				95%CI	
Age (years)	0.975	0.946-1.006	0.112				
BMI (kg/m²)	1.071	1.018-1.127	**0.00**	0.070	1.07	1.019-1.128	**0.007**
WHR	14.19	1.010-199.315	**0.04**	0.783	2.18	0.080-	0.643
Time of	0.914	0.854-0.978	**0.01**	–	0.91	0.853-0.978	**0.009**
Anti-sperm	0.905	0.583-1.406	0.65				
FSH (IU/l)	0.975	0.899-1.057	0.53				
T (nmol/l)	0.995	0.938-1.056	0.87				
LH(IU/l)	0.940	0.845-1.046	0.25				
Alcohol	1.117	0.915-1.364	0.27				
Smoke	1.131	0.841-1.520	0.41				

BMI, Body Mass Index; WHR, Waist-Hip Ratio; FSH, Follicular Stimulating Hormone; LH, Luteinizing Hormone; T, Testosterone. Statistically significant data were represented in bold.

**Table 5:** Univariate and multivariate logistic analysis for screening the independent factors of sperm morphology.

Variables	OR	Univariate analysis	*P*	B	OR	Multivariate analysis	*P*
		95%CI				95%CI	
Age (years)	0.987	0.952-1.024	0.48				
BMI (kg/m²)	1.053	0.990-1.121	0.10				
WCR	2.958	0.122-	0.50				
Time of	1.041	0.963-1.125	0.31				
Anti-sperm	1.005	0.591-1.705	0.98				
FSH (IU/I)	0.820	0.723-0.931	**0.00**	–	0.82	0.727-0.927	**0.002**
T (nmol/l)	0.924	0.854-1.000	**0.04**	–	0.90	0.835-0.987	**0.023**
LH(IU/1)	0.884	0.794-0.985	**0.02**	–	0.97	0.844-1.115	0.670
Alcohol	1.233	0.965-1.576	0.095				
Smoke	0.792	0.555-1.130	0.199				

BMI, Body Mass Index; WHR, Waist-Hip Ratio; FSH, Follicular Stimulating Hormone; LH, Luteinizing Hormone; T, Testosterone. Statistically significant data were represented in bold.Statistically significant data were represented in bold.

## 4 Discussion

In this observational study, the association between BMI, WHR, and semen quality was investigated in 715, sperm donors who underwent semen examinations between 2019 and 2021 in Hefei, China. We found that BMI was weakly associated with sperm progressive motility, while WHR showed no relationship with semen parameters. The RCS showed that BMI was negatively associated with sperm concentration while BMI was more than 22.40 kg/m^2^ and the WHR was negatively related to sperm progressive motility within 0.82 to 0.89. Furthermore, the multivariate logistic analysis showed that BMI was an independent risk factor for normal sperm progressive motility, FSH was an independent risk factor for normal sperm concentration and morphology, and testosterone was an independent risk factor for sperm morphology. This study highlights that BMI is a more important factor in the assessment of semen quality than WHR. BMI showed a weak influence on sperm progressive motility; however, serum FSH and testosterone were the main factors affecting sperm concentration and morphology.

The relationship between BMI and semen quality remains controversial. Some studies have demonstrated that an increased BMI is significantly linked with sperm concentration, sperm motility, and sperm morphology (8, 9, 13). However, other studies reported different conclusions (7, 9, 14). In 2013, Sermondade et al. performed a meta-analysis on a large sample of data (15). They found that being overweight and obesity were significantly associated with an increased risk of azoospermia and oligozoospermia. However, our study showed that BMI was positively associated with sperm progressive motility, but not with sperm concentration or morphology. The results of the multivariate logistic analysis showing that BMI was an independent risk factor confirmed this. Therefore, an elevated BMI may be beneficial for sperm progressive motility.

Few studies have focused on the relationship between being underweight and semen quality. In 2004, Jensen et al. assessed military readiness in 1558.00 young Danish men young men between 1996 and 1998; a significant reduction in sperm concentration was observed in underweight men, which was defined as BMI <20.00 kg/m^2^ (16). A later study by Qin et al. (17) reported that being underweight was significantly associated with a lower sperm concentration in fertile men from the general population. Consistent with these two studies, being underweight was inversely related to sperm concentration and total sperm number, but not to sperm motility or semen volume, in Jixuan Ma’s study, which recruited 3966.00 healthy sperm donors (9). In the present study, an increased prevalence of azoospermia was observed in the underweight group and the sperm concentration was lower compared with the normal bodyweight or overweight group. This was consistent with previous research. Malnutrition may be the key factor of the association between being underweight and worse semen quality, which is known to have negative effects on male reproductive hormones (18).

It is well known that reproductive hormones, including FSH and testosterone, are closely related to male semen quality (19–21). FSH plays an important role in spermatogenesis. Clinical studies reported that FSH levels were higher in infertile patients compared with fertile controls, and that FSH was negatively correlated to sperm concentration in the population surveyed (22). However, the relationship between FSH and sperm morphology has been rarely reported. In 2020, Wei Zhao et al. (23) reported that FSH was inversely associated with sperm morphology after mutual adjustment, which was consistent with our finding. This indicated that FSH is important for sperm to maintain normal morphology. However, the mechanism remains to be studied in the future. In addition, testosterone is known to be the key factor to sustain sperm production. Therefore, testosterone may be strongly associated with sperm concentration, sperm morphology, or sperm motility. As reported, using human chorionic gonadotropin or tamoxifen citrate in infertile men with testosterone deficiency led to an improvement of the sperm concentration, sperm motility, and sperm morphology (24). Nevertheless, the results of the present study showed that testosterone was an independent risk factor for sperm morphology but was not related with sperm concentration or sperm morphology. A possible explanation is that the levels of testosterone in the serum in the present study did not reflect the true intratesticular testosterone level, which is believed to directly act in the spermatogenic process.

There are some limitations in the present research. Firstly, the sample size collected was small, especially for people who had completed sex hormone tests. Secondly, the data was acquired in the infertile population, whose age were concentrated in the reproductive age. Therefore, the result of this study cannot represent the result of all populations. Thirdly, this is a single-center retrospective research, the conclusion needs to be verified by a multi-center prospective study in the future.

In conclusion, BMI is an independent risk factor for sperm motility while the WHR did not contribute to semen parameters. Our findings highlight the important role of serum FSH and testosterone in male semen quality.

## Data availability statement

The original contributions presented in the study are included in the article/supplementary material. Further inquiries can be directed to the corresponding authors.

## Author contributions

XH and SW designed the research study. ZK, CW and PX contributed to the data acquisition. XH, BW and JX analyzed the data. SW wrote the paper. All authors approved the final manuscript.
